# The Neural Signals of the Superior Ovarian Nerve Modulate in an Asymmetric Way the Ovarian Steroidogenic Response to the Vasoactive Intestinal Peptide

**DOI:** 10.3389/fphys.2018.01142

**Published:** 2018-08-20

**Authors:** Gabriela Rosas, Rosa Linares, Deyra A. Ramírez, Elizabeth Vieyra, Angélica Trujillo, Roberto Domínguez, Leticia Morales-Ledesma

**Affiliations:** ^1^Biology of Reproduction Research Unit, Physiology of Reproduction Laboratory, Facultad de Estudios Superiores Zaragoza, Universidad Nacional Autónoma de Mexico, Mexico City, Mexico; ^2^Benemérita Universidad Autónoma de Puebla, Facultad de Ciencias Biológicas, Puebla, Mexico

**Keywords:** vasoactive intestinal peptide (VIP), superior ovarian nerve (SON), estrous cycle, asymmetries, progesterone, testosterone, estradiol

## Abstract

The superior ovarian nerve (SON) provides neuropeptide-Y, norepinephrine and vasoactive intestinal peptide (VIP) to the ovaries. Ovarian steroidogenesis is modulated by the SON. In the cyclic rat, the acute steroidogenic response to ovarian microinjection of VIP is asymmetric and varies during the estrous cycle. In the present study, we analyze whether the differential effects of VIP in each ovary are modulated by the neural signals arriving through the SON. Cyclic female rats were submitted on diestrus-1, diestrus-2, proestrus, or estrus to a unilateral section of the SON, and immediately afterward, the denervated ovary was either microinjected or not with VIP. Animals were sacrificed 1 h after treatment. The injection of VIP into the left denervated ovary performed on diestrus-1 decreased progesterone levels in comparison with the left SON sectioning group; similar effects were observed on proestrus when VIP was injected into either of the denervated ovaries. Compared to the left SON sectioning group, VIP treatment into the left denervated ovary on diestrus-2 or proestrus decreased testosterone levels, whereas on diestrus-1, proestrus or estrus, the same treatment resulted in higher estradiol levels. Compared to the right SON sectioning group, VIP injected into the right denervated ovary yielded higher testosterone levels on diestrus-1 and estrus and lower testosterone levels on proestrus. VIP injection into the right denervated ovary increased estradiol levels on diestrus-2 or estrus while decreasing them on proestrus. Our results indicate that in the adult cyclic rat, the set neural signals arriving to the ovaries through the SON asymmetrically modulate the role of VIP on steroid hormone secretion, depending on the endocrine status of the animal. The results also support the hypothesis that the left and right ovary respond differently to the VIPergic stimulus.

## Introduction

The function and control of endocrine-paired organs are not identical (Klein and Burden, 1988; Frankel et al., 1989; Morán et al., 2005; Tóth et al., 2007; Gerendai et al., 2009; Domínguez and Cruz-Morales, 2011). In the rat, the left and right ovary have different capacities to release steroid hormones, a response that depends on the day of the estrous cycle studied (Barco et al., 2003; Flores et al., 2005, 2006; Cruz et al., 2006). Regarding the spontaneous release of oocytes, the left ovary appears to be more competent than the right (Domínguez et al., 1989). Such asymmetries are related to the innervation received by the ovaries (Domínguez et al., 2011); this innervation is different for the left and right ovary (Klein and Burden, 1988; Tóth et al., 2007; Gerendai et al., 2009).

The sympathetic innervation of the mammalian ovary regulates steroidogenesis and ovulation (Aguado, 2002; Lara et al., 2002; Domínguez and Cruz-Morales, 2011). The rat ovary is innervated by two sympathetic sources, the ovarian plexus nerve and the SON (Lawrence and Burden, 1980). The SON originates from the celiac–superior mesenteric ganglia; runs together with the suspensory ligament and predominately innervates steroidogenic cells, particularly the theca and interstitial cells; and provides the ovary with NE, NPY, and VIP (Baljet and Drukker, 1979; Lawrence and Burden, 1980; Burden, 1985; Dees et al., 1986; Klein and Burden, 1988). The SON is the main source of NE (Aguado and Ojeda, 1984b) and VIP to the ovary (Dees et al., 1986; Advis et al., 1989).

According to Forneris and Aguado (2002), the bilateral section of the SON to 4-day-old rats delays the onset of puberty, alters the pattern of the estrous cycle, increases the serum estradiol levels and decreases the follicle stimulating hormone, without apparent changes in the luteinizing hormone levels. Zhang et al. (2010) performed the same treatment to 2-day-old rats and observed a lower total number of follicles, a decrease in the ovulatory response to gonadotrophins, and an increase in follicular atresia. These results suggest that interrupting ovarian innervation at an early age has critical effects in ovarian prepubertal development regulation and in the ovarian cyclic activity when rats reach adulthood (Forneris and Aguado, 2002).

In the prepubertal rat, the bilateral section of the SON causes a drop in ovarian NE content (Aguado and Ojeda, 1984b; Chávez et al., 1994). In the adult rat, the bilateral section of the SON performed during proestrus decreased progesterone and estradiol levels in the ovarian vein, an effect that does not occur if the denervation is performed on the day of estrus (Aguado and Ojeda, 1984a). Previously, we showed that in the prepubertal rat, the effects of unilateral section of the SON on steroid hormone levels were asymmetric and depend on the hormone analyzed and the elapsed time between surgery and autopsy (Morales-Ledesma et al., 2012). In the adult rat, the SON modulates steroid hormones secretion in an asymmetric way, depending on the hour of day (Ramírez et al., 2017) and the day of the estrous cycle (Flores et al., 2011).

In the rat, VIP ovarian levels vary throughout the estrous cycle, with the highest level on diestrus-2 (Parra et al., 2007). VIP binds to VPAC1 and VPAC2, which are G protein-coupled receptors (Groneberg et al., 2006; Onoue et al., 2008; Couvineau et al., 2012). In the ovary, VIP activates the adenylate cyclase/cyclic 3′,5′-adenosine monophosphate/protein kinase A pathway (Davoren and Hsueh, 1985; Trzeciak et al., 1986; Gräs et al., 2000; Kowalewski et al., 2010). The theca/interstitial cells of the ovary express both VIPergic receptors, while granulosa cells only express VPAC2 (Vaccari et al., 2006).

Vasoactive intestinal peptide stimulates progesterone, androgen and estradiol release from whole ovary or granulosa cells *in vitro* (Davoren and Hsueh, 1985; Ahmed et al., 1986; Parra et al., 2007). Adding NPY, NE, VIP, or NE plus VIP to the hemiovaries of rats on diestrus-1 decreases progesterone release, while the same treatment on diestrus-2 increases it (Garraza et al., 2004). In cultured ovarian granulosa cells, progesterone secretion stimulated by VIP is potentiated by an α1-adrenergic agonist (Wasilewska-Dziubiñska et al., 2002).

Previously we showed that one or 24 h after microinjecting VIP into the left or right ovary, steroid hormone levels were different depending on the treated ovary, the day of the estrous cycle and the time elapsed between treatment and autopsy. We suggest that “the ovarian asymmetric response to VIP is modulated by the innervation received by each ovary” (Rosas et al., 2015). This study aimed to test the hypothesis that ovarian neural signals arriving through the SON regulate in a different way each ovary’s acute steroidogenic response to the VIP during the estrous cycle. For this purpose, rats on the fourth days of the estrous cycle were submitted to the unilateral section of the SON, followed by a microinjection of VIP into the bursa of the denervated ovary. The effects on steroid hormone levels were evaluated after 1 h.

## Materials and Methods

The experiments were performed in strict accordance with the Mexican Law of Animal Treatment and Protection Guidelines and followed the Mexican Official Standard NOM-062-ZOO-1999 specifications. The experimental protocols used in this study were approved by the Bioethics Committee of the Facultad de Estudios Superiores-Zaragoza, Universidad Nacional Autónoma de México. All possible efforts were made to minimize the number of animals used and their suffering.

Sixty-day-old virgin adult female rats of the CIIZ-V strain from our own stock were used in this study. They were allowed free access to food (Purina S.A., Mexico) and tap water and were housed in acrylic cages under controlled lighting (lights on from 05:00 to 19:00 h) and temperature (22 ± 2°C) conditions. Following the methodology of Marcondes et al. (2002), estrous cyclicity was monitored by daily cytological examinations. In brief, daily vaginal smears were performed every morning at 09:00 h with a sterile inoculating loop; the vaginal cells were placed on a standard slide, stained with hematoxylin-eosin, and observed under a light microscope. Only animals exhibiting at least two consecutive 4-day estrous cycles were used in this study. The surgery treatment was performed between 10:30 and 11:30 h on diestrus-1, diestrus-2, proestrus or estrus. All rats were anesthetized with ether. The animals were sacrificed by decapitation 1 h after the surgical procedure.

### Experimental Groups

Groups of 10 rats were randomly allotted to one of the following experimental groups:

#### Control Group

Groups of cyclic-untouched rats sacrificed on the day of diestrus-1, diestrus-2, proestrus or estrus.

#### Unilateral Sham Surgery

The sham surgery procedure was performed following a previously described methodology (Morales-Ledesma et al., 2011, 2012; Ramírez et al., 2017). In brief, a dorso-lateral incision on the left or right side of the abdominal wall (L-Sham or R-Sham) was performed 2 cm below the last rib. Subsequently, the wound was sutured. The incision affected the skin, muscle and peritoneum, and no organs were manipulated.

#### Unilateral Section of the SON

The section of the left or right SON (L-SON or R-SON, respectively) was performed according to previously described methodology (Morales-Ledesma et al., 2010, 2012; Ramírez et al., 2017). In brief, the anesthetized animals were submitted to a dorso-lateral incision as described above. Then, the ovary was pulled out of the abdominal cavity and, with the aid of small scissors, the suspensory ligament (enclosing the SON) was sectioned at approximately 1 cm from the ovary. The denervated ovary was immediately returned to the abdominal cavity and the wound was sutured.

#### Unilateral Section of the SON Plus VIP Injection Into the Denervated Ovary

Immediately after sectioning the left or right ovary’s SON, VIP was injected into the ovarian bursa, following the methodology previously described (Rosas et al., 2015). In brief, using a 0.5 mL syringe with a 31G × 8 mm gauge needle, were microinjected 20 μL of VIP 10^−6^ M solution (Sigma Chem. Co., St. Luis, MO, United States) into the bursa of the left or right denervated ovary (L-VIP or R-VIP). After VIP solution injection, the needle was kept in the ovarian bursa for one min to prevent leakage and allow it to completely cover the ovary. Then, the denervated ovary was carefully returned to the abdominal cavity and, subsequently, the wound was sutured.

### Autopsy Procedures

At the autopsy, trunk blood was collected, allowed to clot at room temperature for 30 min and was centrifuged at 3,000 rpm for 15 min. The serum was separated and stored at −20°C until progesterone, testosterone and estradiol levels were measured. During autopsy, the denervation of the ovary was confirmed by the free movement of the ovary in the abdominal cavity.

### Hormone Assay

Progesterone, testosterone, and estradiol serum levels were measured by specific radioimmunoassay with kits purchased from Diagnostic Products (Los Angeles, CA, United States). The levels of progesterone are expressed in ng/mL, and testosterone and estradiol in pg/mL. The intra- and inter-assay coefficients of variation were 6.58 and 7.42% for progesterone, 7.85 and 8.76% for testosterone and 7.54 and 8.21% for estradiol, respectively.

### Statistical Analyses

The results in each experimental group are expressed as the mean ± standard error of the mean (SEM). Differences in steroid hormone levels between the two groups were analyzed using the Student’s *t*-test. A multivariate analysis of variance (MANOVA) followed by Tukey’s test was used to perform several comparisons. A value of *P* ≤ 0.05 was considered statistically significant.

## Results

### Effects of Unilateral Sham Surgery on Steroid Hormones Serum Levels

Compared to the control group, the unilateral sham surgery increased progesterone levels on any day of the estrous cycle. The effects of L-Sham or R-Sham surgery on testosterone and estradiol levels depended on the estrous cycle day and the side of the sham surgery (**Table 1**).

**Table 1:** Means ± SEM of progesterone, testosterone and estradiol serum levels in control rats and animals with left or right sham surgery (L-Sham or R-Sham) performed to 10:30 h on diestrus-1, diestrus-2, proestrus, or estrus, sacrificed 1 h later.

Groups	Diestrus-1	Diestrus-2	Proestrus	Estrus
**Progesterone (ng/mL)**				
Control	15.7 ± 0.7	4.2 ± 0.7	5.5 ± 0.7	4.6 ± 0.3
L-Sham	44.4 ± 2.7 **a**	28.1 ± 1.8 **a**	34.0 ± 2.8 **a**	32.0 ± 1.3 **a**
R-Sham	40.2 ± 3.2 **a**	28.5 ± 1.9 **a**	36.7 ± 1.8 **a**	36.9 ± 1.6 **ab**
**Testosterone (pg/mL)**				
Control	50.8 ± 7.2	44.7 ± 2.7	112.9 ± 13.9	30.9 ± 5.2
L-Sham	46.1 ± 2.4	60.7 ± 8.2	206.2 ± 10.3 **a**	68.1 ± 9.6 **a**
R-Sham	42.2 ± 1.4	129.5 ± 12.6 **ab**	238.6 ± 19.3 **a**	16.1 ± 1.2 **b**
**Estradiol (pg/mL)**				
Control	27.2 ± 3.5	72.4 ± 12.8	103.9 ± 15.8	52.2 ± 4.4
L-Sham	53.1 ± 2.6 **a**	97.7 ± 8.7	198.2 ± 7.6 **a**	23.7 ± 2.3 **a**
R-Sham	49.0 ± 4.8 **a**	331.0 ± 35.0 **ab**	127.0 ± 8.5 **b**	45.3 ± 2.2 **b**

***^a^**p < 0.05 vs. control; **^b^**p < 0.05 vs. L-Sham (MANOVA followed by Tukey’s test). The control data groups (diestrus-1, diestrus-2, proestrus, and estrus) were taken from Rosas et al. (2015).*

**FIGURE 1:**
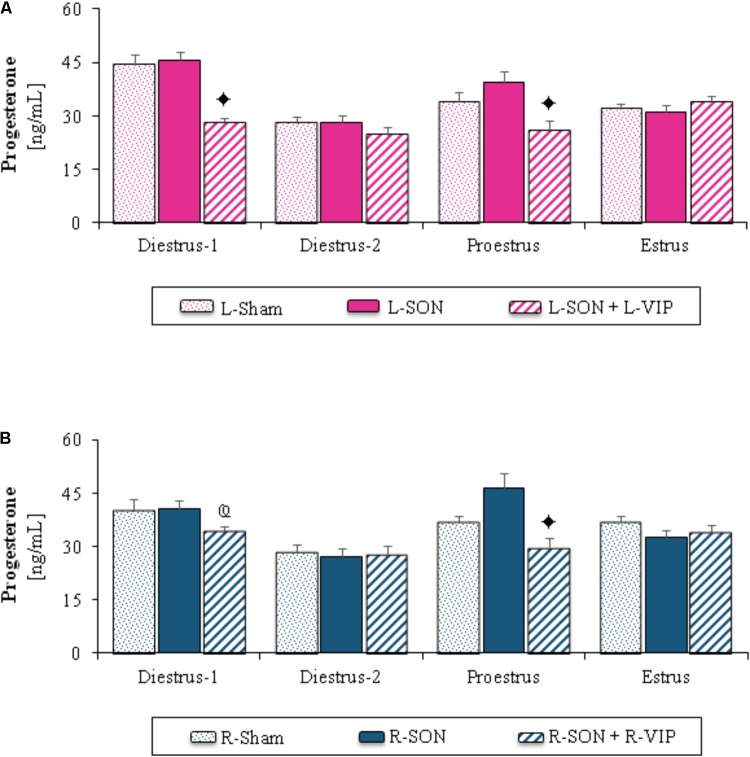
Mean ± SEM of progesterone (ng/mL) serum levels in animals submitted to left **(A)** or right **(B)** sham surgery (L-Sham or R-Sham), or the left or right sectioning of the superior ovarian nerve (L-SON or R-SON) with or without subsequent treatment with VIP into the left or right denervated ovary (L-VIP or R-VIP). Animals were sacrificed 1 h after surgery. 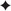
*p* < 0.05 vs. its respective L-SON or R-SON group; 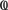
*p* < 0.05 *vs*. group with the same treatment of the left side (Student’s *t*-test).

**FIGURE 2:**
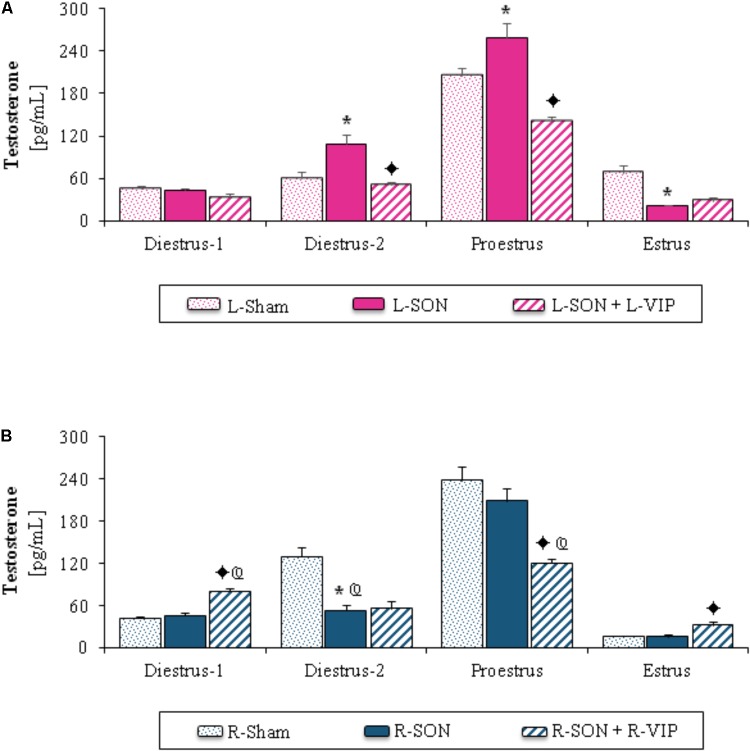
Mean ± SEM of testosterone (pg/mL) serum levels in animals submitted to left **(A)** or right **(B)** sham surgery (L-Sham or R-Sham), or the left or right sectioning of the superior ovarian nerve (L-SON or R-SON) with or without subsequent treatment with VIP into the left or right denervated ovary (L-VIP or R-VIP). Animals were sacrificed 1 h after surgery. **^∗^***p* < 0.05 vs. its respective L-Sham or R-Sham group; 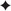
*p* < 0.05 vs. its respective L-SON or R-SON group; 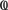
*p* < 0.05 vs. group with the same treatment of the left side (Student’s *t*-test).

**FIGURE 3:**
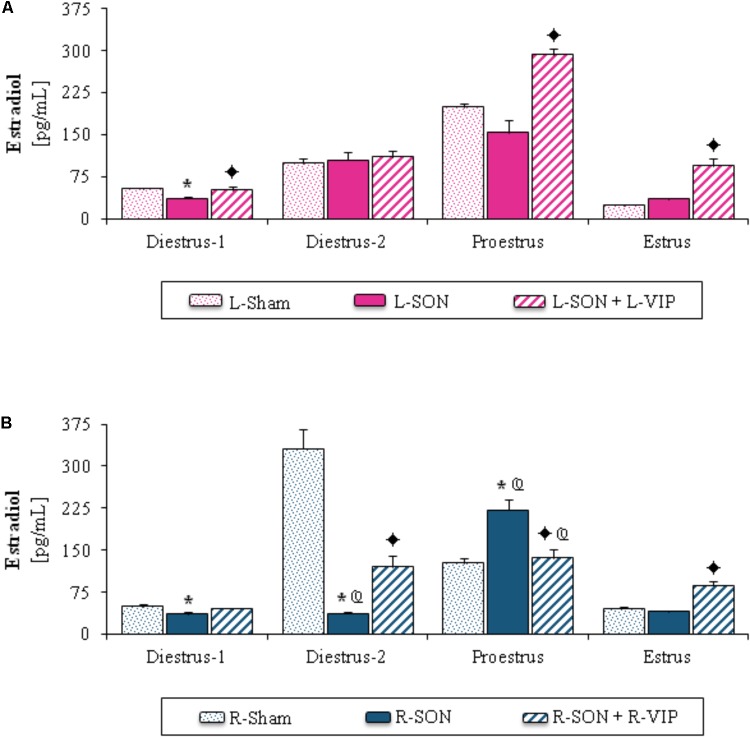
Mean ± SEM of estradiol (pg/mL) serum levels in animals submitted to left **(A)** or right **(B)** sham surgery (L-Sham or R-Sham), or the left or right sectioning of the superior ovarian nerve (L-SON or R-SON) with or without subsequent treatment with VIP into the left or right denervated ovary (L-VIP or R-VIP). Animals were sacrificed 1 h after surgery. ^∗^*p* < 0.05 vs. its respective L-Sham or R-Sham group; 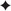
*p* < 0.05 vs. its respective L-SON or R-SON group; 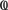
*p* < 0.05 vs. group with the same treatment of the left side (Student’s *t*-test).

Because the steroid hormone levels were modified by the effect of unilateral sham surgery, the results of the L-SON or R-SON (**Figures 1**–**3**) were compared with their respective sham surgery groups.

### Effects of Unilateral Section of the SON or Unilateral VIP Injection Into the Denervated Ovary of Cyclic Rats

#### Progesterone Serum Levels

##### Unilateral section of the SON

The L-SON or R-SON did not modify progesterone levels in comparison to their respective unilateral sham surgery group, independently of the estrous cycle day (**Figures 1A,B**).

##### Unilateral VIP injection into the denervated ovary

The stimulation of the left ovary with VIP on diestrus-1 or the any ovary on proestrus day yielded lower progesterone levels than in those of their corresponding groups with unilateral section of the SON. On diestrus-1, progesterone levels were higher in the group with VIP injection into the right ovary than in the group with VIP injection into the left ovary (**Figures 1A,B**).

#### Testosterone Serum Levels

##### Unilateral section of the SON

The L-SON on diestrus-2 or proestrus yielded higher testosterone levels than in L-Sham and lower ones at estrus (**Figure 2A**). On diestrus-2, the R-SON decreased testosterone levels compared to the R-Sham (**Figure 2B**) and L-SON groups (**Figures 2A,B**).

##### Unilateral VIP injection into the denervated ovary

On diestrus-2 or proestrus, the stimulation of the left ovary with VIP yielded lower testosterone levels than in the L-SON group (**Figure 2A**). In rats with VIPergic stimulation of the right ovary performed on diestrus-1 or estrus, testosterone levels were higher than in the R-SON group, while on proestrus they were lower (**Figure 2B**).

In comparison with animals injected with VIP into the left ovary, the injection of VIP into the right ovary of rats on diestrus-1 increased testosterone levels, on proestrus, the same treatment lowered testosterone levels (**Figures 2A,B**).

#### Estradiol Serum Levels

##### Unilateral section of the SON

Compared to the L-Sham group, the L-SON performed on diestrus-1 yielded lower estradiol levels (**Figure 3A**). In rats submitted to R-SON, estradiol levels were lower on diestrus-1 or diestrus-2 than in their corresponding R-Sham group, while on proestrus they were higher (**Figure 3B**). In comparison with L-SON, the R-SON performed on diestrus-2 yielded lower estradiol levels, while they were higher on proestrus (**Figures 3A,B**).

##### Unilateral VIP injection into the denervated ovary

Higher estradiol levels were observed in rats with VIP treatment into the left ovary on diestrus-1, proestrus or estrus than in L-SON groups (**Figure 3A**). On diestrus-2 or estrus day, the VIPergic stimulation of the right ovary yielded higher estradiol levels than in the R-SON group, and lower ones on proestrus (**Figure 3B**).

In comparison with animals injected into the left ovary, the VIP treatment into the right ovary performed on proestrus resulted in lower estradiol levels (**Figures 3A,B**).

## Discussion

Present results show that the neural signals arriving to the ovaries through the SON, such as NE and NPY, modulate the effects of VIP on steroid hormone secretion. Such effects vary during the estrous cycle and depend on the ovary studied.

The ovarian innervation releases neurotransmitters at a specific time during the day. Throughout the estrous cycle, the release amplitudes and frequencies vary, regulating the follicular, luteal and interstitial response to the follicle stimulating hormone and the luteinizing hormone (Domínguez and Cruz-Morales, 2011).

The ovaries and adrenals synthesize progesterone (Domínguez et al., 2011; Louw-du Toit et al., 2017). Progesterone secretion increases under a different nature and duration of stress (Kalil et al., 2013; Kalász et al., 2014; Ariza Traslaviña et al., 2014) and varies according to the stressor used and the day of the estrous cycle when the stress is applied (Barco et al., 2003; Flores et al., 2005; Ariza Traslaviña et al., 2014). Given that during the rat’s estrous cycle, the adrenal glands are the main source of progesterone (Flores et al., 2008), and under stress conditions is the only source of progesterone (Budec et al., 2002; Romeo et al., 2004; Kalil et al., 2013; Kalász et al., 2014), we suggest that the higher progesterone levels found in sham surgery treated animals are due to the stress caused by the perforation of the peritoneum. Although we do not rule out that it is the result of the activation of a neural pathway; Barco et al. (2003) and Uchida et al. (2005) have pointed out that there exists a neural pathway between the peritoneum and the ovaries and the adrenals.

In the present study, the asymmetric effects on testosterone and estradiol secretion in rats submitted to unilateral sham surgery and their variations throughout the estrous cycle were similar to those previously described (Cruz et al., 2006; Flores et al., 2006). In accordance with Flores et al. (2008), “the neural signals arising from different zones of the peritoneum and the abdominal wall play different roles in the mechanisms regulating steroid hormone levels.” Uchida et al. (2005) showed that the “stimulation of the abdominal skin at the right or left side produced an increase in ovarian sympathetic nerve activity.” Then, we suggest that in sham surgery treated rats, the higher testosterone and estradiol levels observed in the present study could result from abdominal skin and/or peritoneum nerve pathways that send information to the ovary through the SON, depending on the estrous cycle day and the side of the abdominal wall.

According to Slominski et al. (2013), the skin has local steroidogenic activity whose contribution of androgens and estrogens can be significant at a systemic level. Therefore, we do not rule out that part of the observed changes in steroid hormone levels due to sham surgery are of cutaneous origin.

Using an *ex vivo* coeliac ganglion–SON–ovary system, it has been shown that NE modulates the expression of P450-aromatase and 3β-hydroxysteroid dehydrogenase, depending on estral-stage-specific ovarian structures (Delgado et al., 2010). Weiss et al. (1982) reported that “the neural control of ovarian steroidogenesis may be either excitatory through the stimulation of β-adrenergic receptors, or inhibitory through the stimulation of α-receptors.” In fact, it has been shown that the electrical stimulation of the SON on estrus day decreases the ovarian testosterone and estradiol secretion (Kagitani et al., 2008; Uchida and Kagitani, 2014), via the activation of different subtypes of α-adrenoreceptors: α1 for testosterone (Uchida and Kagitani, 2014) and α2 for estradiol (Kagitani et al., 2011; Uchida, 2015).

In this and other studies (Flores et al., 2011; Morales-Ledesma et al., 2012; Ramírez et al., 2017) it has been observed that in animals with a unilateral section of the SON, the estradiol secretion does not always correlate with those of its precursors. These results may be due to that the neuronal signals that reach the ovaries through the SON, activates different types of receptors and consequently modulates in a different way the activity of each enzyme involved in the ovarian steroidogenesis process, depending on the day of the estrous cycle.

Previously, we showed that in adult rats, the SON’s modulation of steroid hormone secretion is asymmetric and depends on the day of estrous cycle (Flores et al., 2011). In this study, similar results on testosterone and estradiol secretion were obtained. In addition, the left SON seems more involved in regulating testosterone secretion while the right SON is more involved in regulating estradiol secretion. The different roles of each SON on steroid hormone secretion throughout the estrous cycle may be due to: (1) the type of adrenergic receptors activated in the ovary (Weiss et al., 1982; Kagitani et al., 2008, 2011; Uchida and Kagitani, 2014); (2) the number of active neurons between ovaries and the celiac–superior mesenteric ganglia that, according to Morán et al. (2005), vary along the estrous cycle; (3) different NE release to the ovary from nerve terminals in each estrous cycle day (Ferruz et al., 1991); and (4) the nerve signals that stimulate the somas of the SON, as shown using an *ex vivo* coeliac ganglion-SON-ovary system (Garraza et al., 2004; Delgado et al., 2010).

In culture of granulosa cells or the whole ovary, adding VIP to the medium increased the secretion of progesterone, androgen and estradiol (Davoren and Hsueh, 1985; Ahmed et al., 1986; Garraza et al., 2004; Parra et al., 2007). These effects were associated with an increase in: (a) the expression and phosphorylation of acute steroidogenic regulatory protein (Kowalewski et al., 2010); (b) the synthesis of cholesterol side-chain cleavage enzyme complex (Trzeciak et al., 1986); (c) the 3β-hydroxysteroid dehydrogenase activity (Davoren and Hsueh, 1985); (d) the 17α-hydroxylase mRNA levels (Johnson et al., 1994); and (e) the aromatase activity (George and Ojeda, 1987).

The present study supports our hypothesis that the ovarian response to VIP is modulated by the innervation received by each ovary (Rosas et al., 2015), since the ovaries’ steroidogenic response to VIPergic stimulation is opposite when the neural signals that arrive into the ovary through the SON are removed by its section.

The SON has a stimulatory or inhibitory role on steroid hormone secretion depending on kind of adrenoreceptors activated (Weiss et al., 1982; Kagitani et al., 2008, 2011; Uchida and Kagitani, 2014; Uchida, 2015). Given that ovarian VIP levels vary along the estrous cycle (Parra et al., 2007) and that the physiological effects of VIP are mediated by two VIPergic receptors (Groneberg et al., 2006; Onoue et al., 2008; Couvineau et al., 2012), which are distributed differentially in the follicle (Vaccari et al., 2006), it is possible for the simultaneous activation of adrenergic and VIPergic receptors, to be different during each day of the estrous cycle and, consequently, also in the secretion of steroid hormones. This possibility is supported by the following evidences: (1) the stimulatory effects of VIP on progesterone secretion in granulosa cells culture was potentiated by an α1-adrenergic agonist, but not by a β_2_-adrenergic agonist (Wasilewska-Dziubiñska et al., 2002); and (2) *in vitro* studies showed that in the hemiovaries of rats, VIP has an inhibitory effect on progesterone release on diestrus-1 and a stimulatory effects on diestrus-2, which were amplified by NE only on diestrus-1 (Garraza et al., 2004).

These evidences led us to propose that the NE, the NPY or both neurotransmitters interact with the VIPergic signals and modulate their effects on progesterone, testosterone and estradiol secretion; and appears to be different according to the treated ovary and the estrous cycle day. In addition, we propose that the asymmetric response of the ovaries to VIP is due to a differential expression of the VIPergic receptors in each ovary, which fluctuates according to the day of the estrous cycle. Nevertheless, further study is needed to confirm these hypotheses.

## Conclusion

These results indicate that the ovaries’ steroidogenic response to VIPergic stimulation depends on the neural signals of the SON, is asymmetrical, and varies throughout the estrous cycle. The results also support the idea that “in the adult cyclic rat each ovary has a different sensitivity to VIPergic stimulation” (Rosas et al., 2015).

## Author Contributions

GR, AT, RD, and LM-L designed the study. GR and RL performed the RIA’s to measure the steroid hormone levels. GR, RL, DR, EV, AT, RD, and LM-L participated in the discussion of the results. All the authors read and approved the final manuscript.

## Conflict of Interest Statement

The authors declare that the research was conducted in the absence of any commercial or financial relationships that could be construed as a potential conflict of interest.

## Abbreviations

L-Sham: left sham surgery
L-SON: left section of the SON
L-VIP: injection of VIP into the left denervated ovary
NE: norepinephrine
NPY: neuropeptide Y
R-Sham: right sham surgery
R-SON: right section of the SON
R-VIP: injection of VIP into the right denervated ovary
SON: superior ovarian nerve
VIP: Vasoactive Intestinal Peptide.
